# Functional and Cosmetic Outcomes of Müller Muscle–Conjunctival Resection in Selected Pediatric Ptosis Patients with a Positive Phenylephrine Test

**DOI:** 10.3390/jcm15072551

**Published:** 2026-03-27

**Authors:** Mehmet Goksel Ulas, Merve Emul, Husna Topcu, Ayse Cetin Efe, Fatma Poslu Karademir

**Affiliations:** 1 Department of Ophthalmology, Beyoglu Eye Training and Research Hospital, University of Health Sciences, 34421 Istanbul, Turkey; draysecetin@gmail.com (A.C.E.); fatma.poslu@hotmail.com (F.P.K.); 2 Ercis Sehit Rıdvan Cevik State Hospital, 65400 Van, Turkey; merveemul@outlook.com; 3 Department of Ophthalmology, Aritmi Osmangazi Hospital, 16220 Bursa, Turkey; husnaozturk@gmail.com

**Keywords:** eyelid symmetry, Müller muscle–conjunctival resection, pediatric blepharoptosis, phenylephrine test

## Abstract

**Background/Objectives**: To evaluate margin reflex distance-1 (MRD-1), inter-eyelid symmetry, functional visual parameters, and cosmetic outcomes following Müller Muscle–Conjunctival Resection (MMCR) in selected pediatric patients with mild-to-moderate blepharoptosis, good levator function, and a positive 2.5% phenylephrine test. **Methods**: This retrospective observational study included pediatric patients (<18 years) who underwent MMCR between 2018 and 2023. Surgical indications were based on functional or developmental criteria, including visual axis obstruction, abnormal head posture, significant eyelid asymmetry, or psychosocial concerns, rather than eyelid height alone. Preoperative and postoperative examinations at 1 week, and at 1, 3, and 6 months, included best-corrected visual acuity (BCVA), MRD-1, eyelid symmetry, levator function, lagophthalmos, and ocular surface findings. Outcomes were analyzed separately for unilateral and bilateral cases. Statistical analyses were performed using non parametric tests, with *p* < 0.05 considered statistically significant. **Results**: Fifty patients (55 eyes; mean age 13.16 ± 4.04 years) were included. Mean preoperative MRD-1 increased significantly from 1.83 ± 0.89 mm to 2.97 ± 0.83 mm at 6 months (*p* < 0.001). Postoperative MRD-1 at 6 months showed a significant correlation with the phenylephrine response. In unilateral cases, excellent or satisfactory postoperative symmetry was achieved in 83.6% of eyes. Bilateral cases demonstrated comparable MRD-1 elevation with satisfactory contour and high patient/parent satisfaction. Transient lagophthalmos improved over time. No overcorrection, exposure keratopathy, or significant ocular surface complications were observed. Revision surgery was required in 8.9% of unilateral cases. **Conclusions**: MMCR is a safe and effective option for appropriately selected pediatric patients, providing predictable eyelid elevation, good symmetry, and low complication rates when functional indications are present.

## 1. Introduction

Blepharoptosis is defined as the downward displacement of the upper eyelid and may present unilaterally or bilaterally. The normal level of the upper eyelid is approximately 1–2 mm below the superior limbus. Mild-to-moderate blepharoptosis refers to a measurable reduction in upper eyelid height, quantified by MRD-1, without complete obstruction of the visual axis. In mild blepharoptosis, MRD-1 is 2–3 mm, usually does not block the visual axis and represents mainly a cosmetic concern. In moderate blepharoptosis, MRD-1 is 1–2 mm and the eyelid covers part of the pupil [1,2]. An MRD-1 of approximately 3–4 mm is generally considered within normal limits in many populations, including Asian cohorts. The unilateral form, especially left ptosis, is much more common, occurring in 63–91.5% of cases [3,4]. If childhood drooping of the upper eyelid occurs in the first year of life, it is called congenital ptosis. Most cases of congenital ptosis are present at birth or recognized within the first year of life. Congenital ptosis is typically caused by myogenic dysgenesis of the levator palpebrae superioris muscle. It should be clearly distinguished from mechanical ptosis (e.g., due to eyelid masses), traumatic ptosis, or neurogenic causes such as third nerve palsy, as these entities differ in pathophysiology and management. Upper eyelid drooping seen later in life is less common and is defined as acquired ptosis. The incidence of ptosis (congenital and acquired) is between 0.79 and 1.99 per 10,000 in different populations, and it is more common in boys [5,6].

Management of pediatric ptosis is more challenging than in adults because of difficulties in examination, cooperation, anesthesia requirements, and the risk of amblyopia or abnormal head posture [7]. In addition to functional visual impairment, ptosis may negatively affect psychosocial development, often necessitating early surgical intervention.

Congenital ptosis is usually caused by levator muscle dysgenesis, and the surgical approach may vary depending on the individual. MMCR surgery is an option for mild to moderate blepharoptosis pediatric patients with good levator function and a positive phenylephrine response [8]. Reported success rates of MMCR range 72% to 97%, with high patient satisfaction, good eyelid symmetry and good cosmetic results [9,10]. This procedure is associated with low complication rates and a faster recovery than the other ptosis surgeries [10].

In pediatric ptosis, surgical options are evaluated according to the type of ptosis, age of onset of ptosis, and levator function. Due to the poor levator function in congenital ptosis, MMCR is less commonly the first-line surgical approach; however, it may be an option for pediatric patients with mild-to-moderate ptosis, good levator function and a positive phenylephrine response [8]. However, evidence regarding the outcomes of MMCR specifically in pediatric populations remains limited, and clear guidance on patient selection and functional indications is still lacking. Several studies have evaluated the outcomes of MMCR for ptosis correction; however, most published series primarily involve adult populations or mixed cohorts that include both adults and children. Liu et al. [1] and Ben Simon et al. [11] reported favorable outcomes following MMCR, but their analyses focused mainly on adult involutional ptosis rather than pediatric disease. Similarly, multicenter studies such as the prospective analysis by Leung et al. [12] included heterogeneous age groups and did not specifically address pediatric surgical indications or developmental considerations. In pediatric populations, the available literature is limited to relatively small retrospective series, and many studies combine different surgical techniques or include patients with variable levator function and heterogeneous ptosis etiologies. Moreover, most previous pediatric reports have relied primarily on subjective cosmetic grading without providing a detailed quantitative analysis of postoperative eyelid symmetry.

Therefore, the present study aims to contribute to the existing literature by focusing on a carefully selected pediatric cohort with mild-to-moderate ptosis, good levator function, and a positive phenylephrine test, and by providing a quantitative assessment of inter-eyelid MRD-1 asymmetry improvement together with functional and cosmetic outcome measures.

## 2. Materials and Methods

This retrospective observational study included pediatric patients who underwent MMCR for the correction of blepharoptosis between 2018 and 2023 at the Oculoplasty Department of our institution. The study was conducted in accordance with the tenets of the Declaration of Helsinki and was approved by the Ethics Committee of Istanbul Training and Research Hospital (Approval date: 27 October 2023, Decision Number: 287). Written informed consent was obtained from the parents or legal guardians of all patients prior to surgery. Age-appropriate assent was obtained from children whenever possible. Written informed consent for publication of clinical photographs was obtained from all patients and their legal guardians. The inclusion criteria were as follows: good levator function (>7 mm), a favorable response to 2.5% phenylephrine hydrochloride testing, pediatric population (age < 18 years), patients without strabismus or requiring surgery and with no history of previous eyelid surgery. Patients with incomplete medical records and previous ptosis surgery were excluded.

Surgery was indicated in pediatric patients with mild-to-moderate ptosis who met at least one of the following functional or clinical criteria: partial obstruction of the visual axis or reduced superior visual field documented on clinical examination, compensatory frontalis overaction or abnormal head posture, inter-eyelid asymmetry ≥ 1.5 mm causing noticeable cosmetic imbalance, or psychosocial concerns reported by patients or parents, including peer-related distress or reduced self-esteem. Visual field assessment was performed according to the patient’s age and cooperation. In cooperative patients (≥10 years), automated perimetry (Humphrey Visual Field Analyzer) was used when feasible. In younger children, superior visual field limitation was assessed clinically by evaluating eyelid position relative to the pupil center and through observation of compensatory head posture. Formal perimetry was not performed in uncooperative young children. Visual axis involvement was defined as the upper eyelid margin crossing the pupillary light reflex in primary gaze. Surgery was not indicated solely for mild partial coverage without associated functional or developmental risk factors. No patient underwent surgery solely based on MRD-1 value without a functional or psychosocial indication. In children aged 6–10 years, surgical intervention was not performed solely for cosmetic reasons but was considered when ptosis was associated with potential risk factors for visual developmental disturbance. Particular attention was given to the possibility of stimulus deprivation amblyopia resulting from intermittent or persistent obstruction of the visual axis. In addition, ptosis-induced corneal astigmatism and significant eyelid asymmetry associated with abnormal head posture were considered potential contributors to amblyogenic risk.

The risk of amblyopia was evaluated through a comprehensive ophthalmologic assessment, including best-corrected visual acuity (BCVA), cycloplegic refraction, and clinical examination of eyelid position relative to the pupillary axis in primary gaze. In cooperative patients, visual acuity asymmetry between eyes and refractive errors were carefully evaluated to exclude anisometropic amblyopia. In younger or less cooperative children, clinical indicators such as persistent eyelid margin crossing the pupillary light reflex, compensatory chin elevation, or documented superior visual field limitation were used as surrogate markers suggesting potential interference with normal visual development. Importantly, surgery was not performed solely to prevent amblyopia in cases of mild ptosis without documented functional or developmental risk factors.

All participants underwent a comprehensive ophthalmic examination, including BCVA were evaluated using the Snellen chart, while intraocular pressure (IOP, mmHg) was recorded with Goldmann applanation tonometry or estimated by digital palpation when tonometry could not be performed. Dilated fundus examinations were conducted utilizing a 90-diopter lens (Volk Optical Inc., Mentor, OH, USA). Demographic and clinical data recorded included age, sex, follow-up duration, amount of resection, MRD-1, eyelid symmetry, response to phenylephrine hydrochloride (Mydfrin^®^ Alcon Laboratories, Inc., Fort Worth, TX, USA) test, Hering’s response, levator function, Bell’s phenomenon, amount of lagophthalmos, ocular motility and complications. Standardized digital photographs were obtained at baseline and at 1, 3, and 6 months postoperatively. MRD-1 and lagophthalmos were measured using Fiji (ImageJ) software (version 2.3.0; National Institutes of Health, Bethesda, MD, USA) with a 10 mm calibration marker placed on the glabella. All photographic measurements were performed by a single masked investigator who was not involved in the surgical procedures. To assess intra-observer reliability, measurements were repeated in a random subset of 20 eyes two weeks apart, and intraclass correlation coefficients (ICCs) were calculated. The phenylephrine test was performed by instilling 2.5% phenylephrine hydrochloride into the superior conjunctival fornix. MRD-1 was measured 5–10 min after instillation. A positive phenylephrine test was defined as an increase of ≥1.5 mm in MRD-1 following instillation of 2.5% phenylephrine hydrochloride. In unilateral cases, a postoperative inter-eyelid MRD-1 difference of ≤1.0 mm was considered clinically acceptable symmetry. All procedures were performed by experienced oculoplastic surgeons. In unilateral ptosis cases, surgical success was evaluated primarily based on postoperative inter-eyelid symmetry. Inter-eyelid asymmetry was quantitatively defined as the absolute difference between the MRD-1 of the operated eye and the fellow eye. Preoperative and postoperative asymmetry values (in millimeters) were calculated. Improvement in asymmetry was defined as the reduction in absolute inter-eyelid MRD-1 difference between baseline and postoperative month 6. In bilateral ptosis cases, outcomes were assessed based on postoperative MRD-1 values, eyelid contour, and patient or parent satisfaction rather than inter-eyelid comparison. Symmetry outcomes were categorized quantitatively as follows: excellent (≤0.5 mm inter-eyelid MRD-1 difference), satisfactory (0.5–1.0 mm), and poor (>1.0 mm).

Postoperative satisfaction was evaluated at postoperative month 6 using a structured questionnaire administered during follow-up visits. Satisfaction was assessed using a 5-point Likert scale (1 = very dissatisfied, 2 = dissatisfied, 3 = neutral, 4 = satisfied, 5 = very satisfied), addressing overall cosmetic appearance, eyelid symmetry, and perceived functional improvement.

In patients younger than 10 years, satisfaction was reported by parents or legal guardians. In older children, both patient and parent responses were recorded when feasible.

The questionnaire was administered by an independent ophthalmologist who was not involved in the surgical procedures to minimize potential bias.

Detailed measurement procedures and additional methodological information are provided in the Supplementary Materials.

### 2.1. Surgical Technique

A standardized 8–10 mm resection of the Müller conjunctival complex was performed in accordance with the degree of ptosis and the phenylephrine test result. Prior to general anesthetic administration, preoperative markings were performed on the upper eyelid. Local infiltration of 2% lidocaine with epinephrine was used for hemostasis and surgical field control. A 4-0 silk traction suture was placed at the upper lid margin, and the eyelid was everted using a Desmarres retractor to expose the palpebral conjunctiva and the superior tarsal border. The superior tarsal margin was identified and marked. Half of the planned resection distance was measured posteriorly using calipers, and reference points were marked along the medial limbal, central pupillary, and lateral limbal axes. A 6-0 silk suture was passed through the conjunctiva and Müller’s muscle to provide traction.

The Müller conjunctiva complex was then elevated and secured with a Putterman clamp without including the tarsal plate. A 6-0 polypropylene suture (Prolene^®^, Ethicon, Inc., Somerville, NJ, USA) was placed in a full-thickness running fashion beneath the clamp. The tissue was sharply excised between the clamp and the sutures using an 11° blade, and the suture was exteriorized through the skin. The conjunctival edges were closed with a continuous 6-0 polypropylene suture with bolster reinforcement.

Postoperatively, preservative-free artificial tears (Hyonat^®^, Biofarma İlaç San. ve Tic. A.Ş., İstanbul, Turkey) were prescribed four times daily, and tobramycin ophthalmic ointment (Tobrased^®^, Bilim İlaç San. ve Tic. A.Ş., İstanbul, Turkey) was applied three times daily to both the ocular surface and suture sites. Sutures were removed at the first postoperative week.

### 2.2. Statistical Analysis

Statistical analyses were conducted using the IBM SPSS Statistics for Mac version 25.0 (SPSS Inc., Chicago, IL, USA) software package. Descriptive statistics are presented as mean ± standard deviation (SD) for continuous variables, and as frequency and percentage (%) for categorical variables. The normality of the data distribution was assessed using the Shapiro–Wilk test. Since the data did not follow a normal distribution, non-parametric tests were employed. Comparisons between unilateral and bilateral groups were performed using the Mann–Whitney U test for continuous variables (e.g., age, levator function, MRD-1 values, lagophthalmos) and the Fisher’s exact test for categorical variables (e.g., sex distribution, symmetry grades, complications, revision rates), given the small number of bilateral cases. Changes in MRD-1 over time (preoperative, postoperative 1 week, 1 month, 3 months, and 6 months) were analyzed using the Friedman test. When a significant overall difference was detected, post hoc pairwise comparisons were conducted using the Wilcoxon signed-rank test with Bonferroni correction. Correlation between postoperative MRD-1 and phenylephrine response was analyzed using Spearman’s rank correlation test.

Symmetry-based outcome classification (excellent/satisfactory/poor) was applied only to unilateral ptosis cases. Bilateral cases were evaluated using absolute postoperative MRD-1 elevation, eyelid contour, and patient/parent satisfaction rather than inter-eyelid comparison. Analyses were primarily conducted at the eye level. Sensitivity analyses excluding the second eye from bilateral patients demonstrated similar trends, minimizing the impact of inter-eye dependence. Changes in inter-eyelid asymmetry were analyzed using the Wilcoxon signed-rank test. The magnitude of asymmetry improvement was also expressed as median change (Δ mm). Photographic MRD-1 and lagophthalmos measurements obtained with ImageJ/Fiji were performed by a masked investigator. Intra-observer reliability was evaluated using the intraclass correlation coefficient (ICC).

To explore potential predictors of surgical success, unilateral cases were stratified according to postoperative symmetry outcomes (excellent/satisfactory vs. poor) and revision surgery requirement. Preoperative clinical variables, including age, sex, baseline MRD-1, levator function, degree of preoperative inter-eyelid asymmetry, and phenylephrine response, were compared between outcome groups using the Mann–Whitney U test or Fisher’s exact test, as appropriate.

A two-tailed *p* value < 0.05 was considered statistically significant.

## 3. Results

A total of 55 eyes from 50 patients (29 females, 21 males) were included. The mean age was 13.16 ± 4.04 years (range, 6–17 years), and the mean preoperative levator function was 13.62 ± 2.95 mm (Table 1). The underlying etiology was congenital myogenic ptosis in 42 patients (84%), and mild acquired aponeurotic ptosis in 8 patients (16%). Among the eight patients with acquired aponeurotic ptosis, the presumed underlying causes included chronic eye rubbing associated with allergic conjunctivitis (*n* = 4), prolonged contact lens use (*n* = 1), and idiopathic aponeurotic dehiscence without a clearly identifiable cause (*n* = 3). None of the patients had a history of eyelid trauma, prior ocular surgery, or systemic neuromuscular disorders. These cases presented with mild ptosis and good levator function, which is consistent with aponeurotic pathology rather than myogenic dysfunction. Because the number of acquired cases was limited and their clinical characteristics were similar to the congenital group in terms of levator function and phenylephrine response, they were analyzed together within the overall cohort. Bilateral surgery was performed in five patients (10 eyes). Because a limited number of patients underwent bilateral surgery (5 patients, 10 eyes), analyses were primarily descriptive at the eye level. Sensitivity analyses excluding the second eye demonstrated similar trends and did not materially change the results. BCVA remained stable, with no significant difference between preoperative and final measurements.

Mean preoperative MRD-1 was 1.83 ± 0.89 mm. Postoperative MRD-1 values increased to 2.35 ± 0.96 mm at 1 week, 2.90 ± 0.91 mm at 1 month, 3.07 ± 0.79 mm at 3 months, and 2.97 ± 0.83 mm at 6 months. The mean MRD-1 after the 2.5% phenylephrine hydrochloride test was 3.56 ± 0.85 mm. Postoperative MRD-1 at 6 months demonstrated a significant positive correlation with the phenylephrine-induced MRD-1 elevation (Spearman correlation coefficient (r = 0.524), *p* < 0.001). Longitudinal analysis using the Friedman test demonstrated a significant change across follow-up visits (*p* < 0.001). Post hoc Wilcoxon signed-rank tests with Bonferroni correction confirmed that MRD-1 at each postoperative visit was significantly higher than baseline (*p* < 0.001 for all comparisons). When stratified by laterality, both unilateral and bilateral groups demonstrated comparable and sustained improvement in eyelid height (Table 2). In unilateral cases, the mean preoperative inter-eyelid MRD-1 difference was 2.13 ± 1.08 mm, which significantly decreased to 0.75 ± 1.09 mm at postoperative month 6. The mean asymmetry improvement was 1.38 mm, representing a large effect size (r ≈ 0.53) (Table 3).

When unilateral cases were stratified according to symmetry outcome, no statistically significant differences were observed in age, sex distribution, or baseline levator function between patients with excellent/satisfactory results and those with poor symmetry (*p* > 0.05). However, patients with poor postoperative symmetry demonstrated a greater mean preoperative inter-eyelid asymmetry and a lower phenylephrine response compared with those achieving favorable outcomes, although these differences did not reach statistical significance. Similarly, cases requiring revision surgery tended to have lower preoperative MRD-1 values and larger baseline asymmetry, suggesting that more severe initial asymmetry may be associated with a higher likelihood of undercorrection.

Throughout the follow-up period, no new cases of amblyopia were detected, and visual acuity remained stable in all patients.

Transient postoperative lagophthalmos averaged approximately 1.5 mm in the early postoperative period and decreased to 0.7 mm by 6 months. No cases of overcorrection, exposure keratopathy, or clinically significant ocular surface complications were observed. Additionally, no contralateral ptosis related to Hering’s phenomenon developed after surgery. Among unilateral cases, postoperative eyelid symmetry was classified as excellent in 27 eyes (60.0%), satisfactory in 12 (26.7%), and poor in 6 (13.3%). As predefined, symmetry-based classification was not applied to bilateral cases; instead, these patients demonstrated satisfactory absolute MRD-1 elevation, symmetric contour, and high patient/parent satisfaction. Undercorrection was the most common complication, occurring in 7 unilateral eyes (15.6%) and 1 bilateral eye (10.0%). No cases of overcorrection or exposure keratopathy were recorded. Revision levator surgery was recommended in five patients; one declined further treatment, and four underwent successful revision surgery. The majority of patients/parents (≥85%) reported being satisfied or very satisfied (Likert score ≥ 4) (Table 4).

## 4. Discussion

In this retrospective study, we evaluated the surgical outcomes of MMCR in carefully selected pediatric patients with unilateral or bilateral ptosis who demonstrated a positive response to phenylephrine hydrochloride. Our findings indicate that MMCR leads to significant and predictable improvement in MRD-1, high rates of postoperative eyelid symmetry, and a low incidence of complications. These results support MMCR as a reliable and effective surgical option in appropriately selected pediatric cases. Although the mean preoperative MRD-1 values suggested mild ptosis, surgical indications were not based solely on eyelid height. Instead, functional, developmental, and psychosocial considerations—including visual axis obstruction, abnormal head posture, and cosmetic asymmetry—guided the decision for intervention. This approach is consistent with contemporary pediatric ptosis management principles. In addition, we acknowledge that symmetry-based outcome classification is primarily applicable to unilateral cases; therefore, bilateral cases were evaluated using absolute eyelid height and cosmetic satisfaction rather than inter-eyelid comparison.

An additional point that warrants clarification is the presence of acquired aponeurotic ptosis in a subset of our pediatric patients. Aponeurotic ptosis is typically associated with aging and is less common in children. However, pediatric aponeurotic dehiscence has been described in association with chronic eye rubbing, allergic conjunctivitis, prolonged contact lens wear, and occasionally idiopathic aponeurotic attenuation. In our cohort, four patients had a history of chronic eye rubbing related to allergic conjunctivitis, one had prolonged contact lens use, and in three patients, no clear precipitating factor could be identified. Importantly, none of these patients had a history of trauma or prior ocular surgery. Although the inclusion of these cases introduces some etiological heterogeneity, all patients demonstrated mild ptosis, good levator function, and a positive phenylephrine response, which represent the primary selection criteria for MMCR. Therefore, these cases were considered clinically appropriate candidates for posterior lamellar surgery and were analyzed together with the congenital cases.

Management of pediatric blepharoptosis remains challenging. Clinical examination may be difficult because of limited cooperation, and measurements of levator function can be variable. Furthermore, the predictive value of the phenylephrine test in children has been reported to be inconsistent [13]. For these reasons, many surgeons remain hesitant to use MMCR in the pediatric population. Nevertheless, our results demonstrate that, in carefully selected patients with good levator function and a positive phenylephrine response, MMCR can achieve favorable outcomes.

Previous studies have reported MMCR success rates ranging from 72% to 95%, with excellent symmetry and high patient satisfaction [9,12]. Symmetry of less than 1 mm can be achieved in most patients after surgery [2,14]. Early reports by Putterman and Fett, as well as Mazow and Shulkin, demonstrated encouraging results in congenital ptosis [15,16]. More recently, Gazit et al. described favorable outcomes using MMCR combined with tarsectomy. Although their technique involved a more aggressive posterior lamellar approach, our findings suggest that MMCR alone can achieve comparable eyelid elevation and symmetry in appropriately selected pediatric patients [17]. Compared with previously published reports, the present study provides several specific contributions to the literature on pediatric ptosis surgery. First, many prior studies evaluating MMCR outcomes have focused primarily on adult involutional ptosis or mixed-age populations, limiting the ability to extrapolate their findings to pediatric patients. Second, several pediatric series include heterogeneous surgical techniques or patients with variable levator function, making it difficult to define clear indications for posterior lamellar surgery. In contrast, our cohort consisted exclusively of pediatric patients with mild-to-moderate ptosis, good levator function, and a positive phenylephrine response, representing a clearly defined surgical indication group. Third, unlike many previous pediatric MMCR studies that rely mainly on categorical cosmetic grading, the present study provides a quantitative analysis of inter-eyelid MRD-1 asymmetry, demonstrating a mean asymmetry reduction of 1.38 mm with a large effect size. This objective measurement offers a more reproducible evaluation of postoperative eyelid symmetry. Finally, our study also incorporates structured patient/parent satisfaction assessment using a standardized Likert-scale questionnaire, providing additional insight into subjective cosmetic outcomes.

Compared with external levator resection, MMCR offers several practical advantages, particularly in children. The absence of a skin incision reduces scarring and eyelid crease irregularities, the operative time is shorter, and postoperative recovery is generally faster. These factors may be particularly beneficial in pediatric patients. Consistent with prior reports, we observed low rates of contour deformities and high postoperative symmetry [18].

Eyelid symmetry is a key determinant of both functional and cosmetic success. The current study demonstrated excellent or satisfactory symmetry in over 83% of patients, consistent with reports describing symmetry < 1 mm in the majority of MMCR surgeries [1,11]. In our study, the proportion of the group defined as having excellent or satisfactory eyelid symmetry is consistent with the symmetry rate in the literature. Undercorrection is the most common complication, with rates generally within 5–15%, while overcorrection is rare [19,20]. Revision surgery for undercorrection is effective, and both re-MMCR and levator resection are viable options [21,22]. Our undercorrection was 16% and no overcorrection was observed. Unlike many previous pediatric MMCR studies that relied primarily on categorical grading, the present study quantitatively demonstrated a significant reduction in inter-eyelid MRD-1 asymmetry, thereby providing an objective assessment of cosmetic symmetry.

In contrast to studies relying solely on informal reporting, satisfaction in the present study was assessed using a structured Likert-scale questionnaire administered by an independent examiner, enhancing the reproducibility and interpretability of subjective outcome measures.

Most studies have found MMCR to be associated with fewer ocular surface problems than levator surgeries, particularly because it preserves anterior lamellar structures and avoids extensive manipulation of the levator complex [18]. According to a study, postoperative dry eye findings after MMCR did not reveal clinically meaningful changes in ocular surface disease parameters [23]. In our cohort, only mild and transient punctate epithelial erosions were observed, and no patient developed persistent exposure keratopathy or clinically significant ocular surface disease.

Amblyopia remains a critical consideration in pediatric ptosis. In cases of mild-to-moderate ptosis, the optical axis can often be opened (e.g., by raising the eyebrow), so amblyopia associated with ptosis is rarely observed in this patient group. The indication of ptosis surgery for amblyopia prevention in children remains controversial. Classical stimulus deprivation amblyopia typically occurs in cases of complete visual axis occlusion and is therefore uncommon in patients with mild-to-moderate ptosis. However, pediatric ptosis may still interfere with visual development through several mechanisms. These include intermittent obstruction of the visual axis, ptosis-induced corneal astigmatism, and abnormal head posture that may disrupt normal binocular visual development.

In the present study, surgery in children aged 6–10 years was not performed solely to prevent classical deprivation amblyopia. Instead, intervention was considered in cases with clinical indicators suggesting potential interference with visual development, including eyelid margin crossing the pupillary light reflex, compensatory chin elevation, or significant eyelid asymmetry. During follow-up, no new cases of amblyopia were detected and visual acuity remained stable. This finding does not contradict the original surgical indication but rather suggests that early surgical correction may eliminate potential amblyogenic factors before permanent visual impairment develops. There is no direct research reporting amblyopia specifically following MMCR surgery in the literature.

From a clinical perspective, our findings suggest that MMCR is most predictable in pediatric patients with mild-to-moderate ptosis, good levator function, and moderate preoperative asymmetry. Although statistical significance was not reached, greater baseline asymmetry and lower phenylephrine responsiveness tended to be associated with less favorable symmetry outcomes and revision surgery. These observations highlight the importance of careful patient selection. In cases with marked asymmetry or borderline phenylephrine responses, surgeons should counsel families regarding the potential risk of undercorrection and the possibility of secondary procedures. Therefore, MMCR appears to be particularly suitable for patients demonstrating robust phenylephrine response and limited preoperative asymmetry. To our knowledge, this study represents one of the few analyses focusing exclusively on a carefully selected pediatric cohort undergoing MMCR with a positive phenylephrine response, and it provides a quantitative assessment of inter-eyelid MRD-1 asymmetry improvement.

This study has several limitations, including its retrospective design, relatively small sample size, and the absence of a direct comparison group with alternative surgical techniques. In addition, most measurements were based on photographic analysis, which may introduce measurement variability despite masking procedures. Prospective, randomized studies with larger pediatric cohorts and longer follow-up are warranted to further define the role of MMCR in this population.

Taken together, these findings provide additional evidence supporting the role of MMCR as a predictable posterior lamellar procedure in carefully selected pediatric patients and highlight the importance of objective symmetry analysis when evaluating cosmetic outcomes.

## 5. Conclusions

MMCR appears to be a safe, effective, and cosmetically favorable surgical option for carefully selected pediatric patients with mild-to-moderate ptosis. The procedure provides predictable eyelid elevation, rapid recovery, and low complication rates, making it a valuable alternative to external levator resection in appropriately chosen cases.

## Figures and Tables

**Table 1:** Demographic and clinical characteristics of the participants.

Characteristic	Unilateral Ptosis (*n* = 45 Patients)	Bilateral Ptosis (*n* = 5 Patients)	Total (*n* = 50)
Gender, *n* (%)			
Female	26 (57.8%)	3 (60.0%)	29 (58%)
Male	19 (42.2%)	2 (40.0%)	21 (42%)
Age (years), mean ± SD	13.2 ± 4.1	13.0 ± 3.9	13.16 ± 4.04
Preoperative levator function (mm), mean ± SD	13.6 ± 3.0	13.5 ± 2.8	13.62 ± 2.95

mm = millimeter, *n* = number, SD = standard deviation.

**Table 2:** MRD-1 Outcomes and Phenylephrine Response in Unilateral vs. Bilateral Ptosis.

	Unilateral Ptosis (*n* = 45)	Bilateral Ptosis (*n* = 10)
	Mean ± SD (mm)	
Preoperative MRD-1	1.81 ± 0.87	1.88 ± 0.92
Postoperative 1 week MRD-1	2.34 ± 0.95	2.38 ± 0.98
	***p*** **^†^ < 0.001**	***p*** **^†^ < 0.001**
Postoperative 1 month MRD-1	2.89 ± 0.92	2.94 ± 0.90
	***p*** **^†^ < 0.001**	***p*** **^†^ < 0.001**
Postoperative 3 months MRD-1	3.05 ± 0.80	3.12 ± 0.77
	***p*** **^†^ < 0.001**	***p*** **^†^ < 0.001**
Postoperative 6 months MRD-1	2.96 ± 0.84	3.02 ± 0.79
	***p*** **^†^ < 0.001**	***p*** **^†^ < 0.001**
Preoperative MRD-1 after phenylephrine HCI	3.58 ± 0.87	3.50 ± 0.82
	***p*** **^†^ < 0.001**	***p*** **^†^ < 0.001**

*p* ^†^: Friedman test. Post hoc pairwise comparisons with Bonferroni correction. Bold values indicate statistical significance. HCI = hydrochloride, MRD-1 = margin reflex distance-1, mm = millimeter, SD = standard deviation.

**Table 3:** Quantitative Analysis of Inter-Eyelid MRD-1 Asymmetry in Unilateral Cases.

	Unilateral Ptosis (*n* = 45)
	Mean ± SD (mm)
Preoperative inter-eyelid MRD-1 difference	2.13 ± 1.08
Postoperative inter-eyelid MRD-1 difference	0.75 ± 1.09
Mean asymmetry improvement	1.38
Effect size (r)	0.53

MRD-1 = margin reflex distance-1, SD = standard deviation.

**Table 4:** Detailed Surgical Outcomes and Clinical Measurements in Unilateral vs. Bilateral Pediatric Ptosis.

	Unilateral Ptosis (*n* = 45)	Bilateral Ptosis (*n* = 10)
	Mean ± SD (mm)	
Lagophthalmos (mm)		
Early postoperative	1.5 ± 0.6	1.4 ± 0.5
Final (6 Months)	0.7 ± 0.4	0.6 ± 0.3
Symmetry outcomes *		Not applicable
Excellent (≤0.5 mm)	27 (60.0%)	-
Satisfactory (0.5–1.0 mm)	12 (26.7%)	-
Poor (>1.0 mm)	6 (13.3%)	-
Complications		
Undercorrection	7 (15.6%)	1 (10.0%)
Overcorrection	0	0
Exposure keratopathy	0	0
Hering phenomenon	0	-
Revision surgery required	4 (8.9%)	0
Postoperative contour	Good-excellent	Symmetric in all
Patient/parent satisfaction (Likert ≥ 4)	High (≥85%)	High (100%)

* Symmetry-based classification was applied only to unilateral ptosis. Between-group comparisons: Mann–Whitney U test for continuous variables; Fisher’s exact test for categorical variables. Bilateral ptosis outcomes were evaluated using absolute MRD-1 elevation, eyelid contour, and satisfaction rather than inter-eyelid comparison.

## Data Availability

The datasets generated and analyzed during the current study are available from the corresponding author upon reasonable request.

## Supplementary Materials

The following supporting information can be downloaded at: https://www.mdpi.com/article/10.3390/jcm15072551/s1, Table S1: Demographic and clinical characteristics of the participants. Table S2: MRD-1 Outcomes and Phenylephrine Response in Unilateral vs. Bilateral Ptosis. Table S3: Quantitative Analysis of Inter-Eyelid MRD-1 Asymmetry in Unilateral Cases. Table S4: Detailed Surgical Outcomes and Clinical Measurements in Unilateral vs. Bilateral Pediatric Ptosis.

## Abbreviations

The following abbreviations are used in this manuscript:

**BCVA**	Best-Corrected Visual Acuity
**GA**	General Anesthesia
**HCl**	Hydrochloride
**ICC**	Intraclass Correlation Coefficient
**IOP**	Intraocular Pressure
**MMCR**	Müller Muscle–Conjunctival Resection
**MRD-1**	Margin Reflex Distance-1
**mm**	Millimeter
**SD**	Standard Deviation

## References

[1] Liu M.T., Totonchi A., Katira K., Daggett J., Guyuron B. (2012). Outcomes of Mild to Moderate Upper Eyelid Ptosis Correction Using Müller’s Muscle–Conjunctival Resection. Plast. Reconstr. Surg..

[2] Dan J., Sinha K.R., Rootman D.B. (2018). Predictors of Success Following Müller’s Muscle-Conjunctival Resection. Ophthalmic Plast. Reconstr. Surg..

[3] Essawy REl Elsada M.A. (2013). Clinical and Demographic Characteristics of Ptosis in Children: A National Tertiary Hospital Study. Eur. J. Ophthalmol..

[4] Berry-Brincat A., Willshaw H. (2009). Paediatric blepharoptosis: A 10-year review. Eye.

[5] Griepentrog G.J., Diehl N.N., Mohney B.G. (2011). Incidence and Demographics of Childhood Ptosis. Ophthalmology.

[6] Nemet A.Y., Segal O., Mimouni M., Vinker S. (2014). Associated morbidity of pediatric ptosis—A large, community based case–control study. Graefe’s Arch. Clin. Exp. Ophthalmol..

[7] O’Donnell B., Codère F., Dortzbach R., Lucarelli M., Kersten R., Rosser P. (2006). Clinical Controversy: Congenital Unilateral and Jaw-Winking Ptosis. Orbit.

[8] Jubbal K., Kania K., Braun T., Katowitz W., Marx D. (2017). Pediatric Blepharoptosis. Semin. Plast. Surg..

[9] Fatani D.R., Kamal Y.F., AlSulaiman H.M. (2025). Müller muscle-Conjunctival Resection (MMCR) Surgery: A Comprehensive Literature Review. Eur. J. Ophthalmol..

[10] Sweeney A.R., Dermarkarian C.R., Williams K.J., Allen R.C., Yen M.T. (2020). Outcomes After Müller Muscle Conjunctival Resection Versus External Levator Advancement in Severe Involutional Blepharoptosis. Am J Ophthalmol..

[11] Ben Simon G.J., Lee S., Schwarcz R.M., McCann J.D., Goldberg R.A. (2007). Müller’s Muscle–Conjunctival Resection for Correction of Upper Eyelid Ptosis. Arch. Facial Plast. Surg..

[12] Leung V.C., Dupuis J.E.K., Ashraf D.C., Idowu O.O., Massicotte E., Vagefi M.R., Kersten R.C., Kalin-Hajdu E. (2023). Müller Muscle Conjunctival Resection: A Multicentered Prospective Analysis of Surgical Success. Ophthalmic Plast. Reconstr. Surg..

[13] Allard F., Durairaj V. (2010). Current techniques in surgical correction of congenital ptosis. Middle East. Afr. J. Ophthalmol..

[14] Golbert M., Pereira F.J., Garcia D.M., Cruz A.A.V. (2016). Contour Symmetry of the Upper Eyelid Following Bilateral Conjunctival-Müller’s Muscle Resection. Aesthet. Surg. J..

[15] Putterman A.M., Fett D.R. (1986). Müller’s muscle in the treatment of upper eyelid ptosis: A ten-year study. Ophthalmic Surg..

[16] Mazow M.L., Shulkin Z.A. (2011). Mueller’s Muscle-Conjunctival Resection in the Treatment of Congenital Ptosis. Ophthalmic Plast. Reconstr. Surg..

[17] Gazit I., Gildener-Leapman J., Or L., Burkat C.N., Pras E., Hartstein M.E. (2019). Müller’s Muscle-conjunctival Resection Combined with Tarsectomy for Treatment of Congenital Ptosis. Ophthalmic Plast. Reconstr. Surg..

[18] Lim H.K., Lau A.Z., Charles W.N., Khajuria A. (2022). Efficacy and Complications of External and Internal Pediatric Blepharoptosis Repair Techniques: A Systematic Review. Ophthalmic Plast. Reconstr. Surg..

[19] Ozturk Karabulut G. (2019). An Alternative Algorithm for Müller Muscle Conjunctival Resection Surgery for Blepharoptosis Management. Beyoglu Eye Journal..

[20] Fechner M., Kappen I.F.P.M., van Rooij J.A.F., van der Lei B. (2024). Posterior Müller Muscle-Conjunctival Resection as a First Step to Treat Eyelid Ptosis: Clinical Results and Treatment Algorithm. Aesthet. Surg. J. Open Forum..

[21] Mangan M.S., Tekcan H., Yurttaser Ocak S., Ozcelik Kose A., Balci S., Ercalik N.Y., Imamoglu S.M. (2023). Müller Muscle–Conjunctival Resection for Treatment of Contralateral Ptosis following Unilateral External Levator Advancement. Plast. Reconstr. Surg..

[22] Karlin J.N., Katsev B., Kapelushnik N., Simon GBen Rootman D.B. (2022). Revision ptosis surgery for under-correction after Müller muscle conjunctival resection. J. Plast. Reconstr. Aesthetic Surg..

[23] Bautista S.A., Wladis E.J., Schultze R.L. (2018). Quantitative Assessment of Dry Eye Parameters After Müller’s Muscle-Conjunctival Resection. Ophthalmic Plast. Reconstr. Surg..

