# Supplementary material for: Functional and Cosmetic Outcomes of Müller Muscle–Conjunctival Resection in Selected Pediatric Ptosis Patients with a Positive Phenylephrine Test
Source: J Clin Med. 2026 Mar 27;15(7):2551. doi: 10.3390/jcm15072551 (PMC13074171; doi:10.3390/jcm15072551)
Supplement: Supplementary file 1 [file jcm-15-02551-s001.zip › Table S1.pdf]

Table S1. Demographic and clinical characteristics of the participants.

**Characteristic**	**Unilateral Ptosis (*n* = 45 patients)**	**Bilateral Ptosis (*n* = 5 patients)**	**Total (*n* = 50)**
Gender, n (%)			
Female	26 (57.8%)	3 (60.0%)	29 (58%)
Male	19 (42.2%)	2 (40.0%)	21 (42%)
Age (years), mean±SD	13.2 ± 4.1	13.0 ± 3.9	13.16 ± 4.04
Preoperative levator function (mm), mean ± SD	13.6 ± 3.0	13.5 ± 2.8	13.62 ± 2.95

mm = millimeter, n = number, SD = standart deviation
