# Supplementary material for: Functional and Cosmetic Outcomes of Müller Muscle–Conjunctival Resection in Selected Pediatric Ptosis Patients with a Positive Phenylephrine Test
Source: J Clin Med. 2026 Mar 27;15(7):2551. doi: 10.3390/jcm15072551 (PMC13074171; doi:10.3390/jcm15072551)
Supplement: Supplementary file 1 [file jcm-15-02551-s001.zip › Table S2.pdf]

Table S2. MRD-1 Outcomes and Phenylephrine Response in Unilateral vs Bilateral Ptosis

	Unilateral ptosis (n = 45)	***Bilateral ptosis (n = 10)***
	**Mean ± SD (mm)**	
Preoperative MRD-1	1.81 ± 0.87	1.88 ± 0.92
Postoperative 1 week MRD-1	2.34 ± 0.95	2.38 ± 0.98
	**$p^{\dagger} < 0.001$**	**$p^{\dagger} < 0.001$**
Postoperative 1 month MRD-1	2.89 ± 0.92	2.94 ± 0.90
	**$p^{\dagger} < 0.001$**	**$p^{\dagger} < 0.001$**
Postoperative 3 months MRD-1	3.05 ± 0.80	3.12 ± 0.77
	**$p^{\dagger} < 0.001$**	**$p^{\dagger} < 0.001$**
Postoperative 6 months MRD-1	2.96 ± 0.84	3.02 ± 0.79
	**$p^{\dagger} < 0.001$**	**$p^{\dagger} < 0.001$**
Preoperative MRD-1 after phenylephrine HCI	3.58 ± 0.87	3.50 ± 0.82
	**$p^{\dagger} < 0.001$**	**$p^{\dagger} < 0.001$**

$p^{\dagger}$: Friedman test. Post-hoc pairwise comparisons with Bonferroni correction. Bold values indicate statistical significance. HCI = hydrochloride, MRD-1 = margin reflex distance-1, mm = millimeter, SD = standard deviation
