# Supplementary material for: Functional and Cosmetic Outcomes of Müller Muscle–Conjunctival Resection in Selected Pediatric Ptosis Patients with a Positive Phenylephrine Test
Source: J Clin Med. 2026 Mar 27;15(7):2551. doi: 10.3390/jcm15072551 (PMC13074171; doi:10.3390/jcm15072551)
Supplement: Supplementary file 1 [file jcm-15-02551-s001.zip › Table S3.pdf]

Table S3. Quantitative Analysis of Inter-Eyelid MRD-1 Asymmetry in Unilateral Cases

	Unilateral ptosis (n=45)
	**Mean ± SD (mm)**
Preoperative inter-eyelid MRD-1 difference	2.13 ± 1.08
Postoperative inter-eyelid MRD-1 difference	0.75 ± 1.09
Mean asymmetry improvement	1.38
Effect size (r)	0.53

MRD-1 = margin reflex distance-1, SD = standard deviation.
