# Supplementary material for: Functional and Cosmetic Outcomes of Müller Muscle–Conjunctival Resection in Selected Pediatric Ptosis Patients with a Positive Phenylephrine Test
Source: J Clin Med. 2026 Mar 27;15(7):2551. doi: 10.3390/jcm15072551 (PMC13074171; doi:10.3390/jcm15072551)
Supplement: Supplementary file 1 [file jcm-15-02551-s001.zip › Table S4.pdf]

Table S4. Detailed Surgical Outcomes and Clinical Measurements in Unilateral vs Bilateral Pediatric Ptosis

	Unilateral ptosis (n = 45)	*Bilateral ptosis (n = 10)*
	**Mean ± SD (mm)**	
Lagophthalmos (mm)		
Early postoperative	1.5 ± 0.6	1.4 ± 0.5
Final (6 Months)	0.7 ± 0.4	0.6 ± 0.3
Symmetry outcomes*		Not applicable
Excellent (≤ 0.5 mm)	27 (60.0%)	-
Satisfactory (0.5-1.0 mm)	12 (26.7%)	-
Poor (> 1.0 mm)	6 (13.3%)	-
Complications		
Undercorrection	7 (15.6%)	1 (10.0%)
Overcorrection	0	0
Exposure keratopaty	0	0
Hering phenomenon	0	-
Revision surgery required	4 (8.9%)	0
Postoperative contour	Good-excellent	Symmetric in all
Patient/parent satisfaction (Likert ≥ 4)	High (> 85%)	High (100%)

*Symmetry-based classification was applied only to unilateral ptosis.

Between-group comparisons: Mann–Whitney U test for continuous variables; Fisher’s exact test for categorical variables.

Bilateral ptosis outcomes were evaluated using absolute MRD-1 elevation, eyelid contour, and satisfaction rather than inter-eyelid comparison.
